# Outcomes following Serial Intragastric Balloon Therapy for Obesity and Nonalcoholic Fatty Liver Disease in a Single Centre

**DOI:** 10.1155/2017/4697194

**Published:** 2017-12-26

**Authors:** Vi Nguyen, Jiawei Li, Jaslyn Gan, Paul Cordero, Shuvra Ray, Alessandro Solis-Cuevas, Mai Khatib, Jude A. Oben

**Affiliations:** ^1^Institute for Liver and Digestive Health, University College London, Royal Free Hospital, Rowland Hill Street, London NW3 2PF, UK; ^2^Department of Gastroenterology and Hepatology, Guy's and St Thomas' Hospital, NHS Foundation Trust, Westminster Bridge Rd., London SE1 7EH, UK; ^3^Department of Biological Sciences, University College London, Gower St., Bloomsbury, London WC1E 6BT, UK

## Abstract

**Background:**

The incidence of nonalcoholic fatty liver disease (NAFLD) continues to parallel the rise in obesity rates. Endobariatric devices such as the intragastric balloon (IGB) may provide an alternative treatment option.

**Methods:**

Outcomes following IGB treatment in 135 patients with obesity and NAFLD (mean baseline weight 117.9 kg; BMI 41.7 kg/m^2^; HOMA-IR 3.6) were retrospectively examined. Clinical, anthropometric, and biochemical changes were analysed at six months and after consecutive treatment with two and three serial IGBs.

**Results:**

After six months, significant changes were seen with weight and BMI (mean reductions of 11.3 kg and 4.1 kg/m^2^, resp., *p* < 0.01 for both). Significant improvements were also seen with ALT, GGT, and HOMA-IR, with all changes corresponding with weight loss. Forty-eight patients received two IGBs, and 20 were treated with three serial IGBs. The greatest amount of total weight loss was observed after the first 6 months (mean weight lost 7.4 kg, versus 3.6 kg and 1.9 kg with two and three IGBs, resp.).

**Conclusions:**

IGB therapy is an effective, alternative nonsurgical means for weight loss in the management of obesity and NAFLD over the short term, with greatest outcomes observed after six months. Improvements in insulin resistance and hepatic transaminases correlated with weight change.

## 1. Introduction

Nonalcoholic fatty liver disease (NAFLD) is now the leading cause of chronic liver disease in most affluent and several emerging economies [1]. It is expected that NAFLD will become the main indication for liver transplantation by 2020 [2].

NAFLD is invariably linked with obesity and closely associated with other complications of the metabolic syndrome (impaired glucose tolerance, hypertension, and dyslipidaemia) [3, 4]. The estimated prevalence of NAFLD largely parallels the obesity epidemic, and although it varies across different regions of the world, the unifying trend is a rapidly increasing rate being observed worldwide [2].

Obesity is primarily caused by an imbalance in energy homeostasis, with nutrient intake exceeding energy expenditure. However, this disequilibrium likely encompasses complex interactions with several other factors, including physical activity levels, genetics, hormonal changes, maternal and perinatal nutrition, and the gut microbiota [5, 7]. The prevalence of obesity, together with its associated metabolic comorbidities, is now recognised as perhaps the most important health pandemic of the 21st century [8, 9].

Although the individual natural history of NAFLD is largely uncertain, up to 30% of patients can progress to develop nonalcoholic steatohepatitis (NASH) [10], with approximately 5% of these patients at further risk of long-term progression to cirrhosis and end-stage liver disease, including hepatocellular cancer [11, 12]. Moreover, the presence of NAFLD is itself associated with a broader range of complications, including a higher risk of cardiovascular disease (CVD) and early mortality, as well as a greater risk of extrahepatic malignancies [13, 14]. The leading cause of morbidity and death among patients with NAFLD/NASH remains adverse cardiovascular events [15].

There is currently no single, reliable treatment for NAFLD/NASH. While several potential therapeutic options are under development, lifestyle interventions remain the most effective treatment modality. Unfortunately, effective weight loss maintenance following lifestyle interventions remains elusive for most, with the majority returning to their baseline weight by 18–24-month follow-up [16, 17].

Weight loss surgeries (such as gastric bypass, sleeve gastrectomy, and gastric banding) have been shown to improve hepatosteatosis, inflammatory changes, and fibrosis in NASH patients and are currently the only treatments that have demonstrated significant, durable weight loss over the longer term [18]. Not all patients, however, are suitable or eligible for bariatric surgery, especially given the higher risk of complications with formal surgery and general anaesthesia, as well as the greater risk of impaired wound healing in obese patients. Interim or definitive endoscopic procedures such as the intragastric balloon (IGB) may therefore offer less-invasive, reversible alternatives to effect weight loss in instances where lifestyle interventions have failed and/or the patient is not a candidate for bariatric surgery.

Several prospective studies have demonstrated the efficacy of IGBs in achieving significant weight loss. Results from a meta-analysis of 15 trials (13 cohort, 2 controlled trials) studying the efficacy of IGBs in 3608 patients reported an average 34% excess weight loss (EWL) in those receiving IGB insertion for 3–6 months, associated with mean body mass index (BMI) reduction of approximately 3.2 kg/m^2^ [19]. A few more, albeit relatively small, studies have also reported associated improvements with features of the metabolic syndrome [20], as well as liver transaminases and indexes of insulin resistance in obese patients with NAFLD over the short-medium term [21, 23].

The aim of this current analysis was to report on the experience of IGB therapy as it is offered for the treatment of obesity and NAFLD. Our primary aim was to examine the efficacy of IGBs, and serial IGB therapy in particular, as an alternative or adjunctive treatment for patients with obesity and insulin resistance, focusing on weight reduction, and associated changes in metabolic and hepatic indices, as well as safety outcomes.

## 2. Materials and Methods

### 2.1. Participants

From 2005 to 2015, 135 obese patients with NAFLD received treatment with the BioEnterics Intragastric Balloon (BIB; Allergan, Goleta, CA, USA) at a single tertiary hospital (Guy's and St Thomas' Hospital, London, UK). Patients were eligible for IGB insertion if they were ≥18 years of age, with a BMI ≥ 27 kg/m^2^. The majority of patients were also insulin resistant at baseline (HOMA-IR score > 2.50), and most had failed previous attempts at weight loss through lifestyle interventions or medical therapy alone. IGB therapy was contraindicated in those with a hiatus hernia > 5 cm, previous gastric surgery, significant gastric erosions or ulceration, >Grade 1 oesophagitis, active coagulopathies (including anticoagulants that could not be withheld), pregnancy, decompensated cirrhosis, contraindications to sedation for endoscopy, and an inability to provide informed consent.

The efficacy of IGB therapy in subjects with a BMI >27–35 kg/m^2^, who may not qualify for formal bariatric surgery, has been demonstrated [24]. A BMI cut-off of >27 kg/m^2^ was therefore chosen for patient selection in this unit.

The presence of NAFLD was determined on the basis of retrospective liver biopsies confirming >5% hepatic steatosis, formal reports of hepatic steatosis on liver ultrasound (performed within six months from the time of IGB insertion), or a raised controlled attenuation parameter (CAP) reading of >268 dB/m on FibroScan-CAP [25].

All experiments were conducted in accordance with the Declaration of Helsinki, and all procedures were carried out with well-informed and written consent.

### 2.2. IGB Insertion

Patients initially underwent gastroscopy under conscious sedation with midazolam and fentanyl. The IGB was inserted orally in deflated form into the stomach and filled under direct endoscopic vision with 500–600 mL of normal saline and 10 mL of methylene blue fluid. At approximately 6 months following insertion, each IGB was removed during another endoscopic procedure during which balloon puncture, fluid removal, and transoral retrieval of the deflated device were undertaken using specifically designed instruments. Both IGB insertion and removal were carried out as day-only procedures, with patients discharged on the same day, provided no complications were encountered. The serial (second and/or third) IGBs were placed approximately 1-2 weeks following removal of the previous balloon, using insertion and removal procedures identical to those described above. Approval for a subsequent IGB was dependent on weight loss achieved with the first balloon, patient tolerance, and preference.

### 2.3. Clinical, Anthropometric, and Biochemical Measurements

Clinical, anthropometric, and biochemical data were examined retrospectively for each patient where available, and differences in outcomes were compared between baseline (prior to the first IGB insertion) and at three separate time points where appropriate: T1; after the removal of the first 1st IGB, T2; after the removal of the 2nd IGB, and T3; after removal of the 3rd IGB. The primary outcome measure was weight loss, with secondary outcome measures including any significant changes in liver function, insulin resistance, and lipid profiles. Safety outcomes were also analysed.

Weight and height measurements were undertaken using standard protocols in the endoscopy suite at baseline (just prior to the IGB insertion procedure) and following IGB removal [26]. Waist circumference was also measured using standard techniques in the outpatient clinics, usually at the time of a follow-up consultation before/after IGB insertion.

Fasting blood samples were collected at baseline and at the time of IGB removal, usually following an outpatient clinic review performed within two weeks before/after the IGB insertion. The following biochemical assays were performed: full blood count, liver function tests, renal function, total cholesterol, high-density lipoprotein (HDL), low-density lipoprotein (LDL), triglycerides, glucose, insulin, HbA1c, vitamin D, and CRP. Serum biochemistry was performed by the diagnostic testing laboratory at St Thomas' Hospital, London, United Kingdom.

Insulin resistance was calculated using the Homeostasis Model Assessment- (HOMA-) IR score, equation: HOMA-IR = [mean fasting insulin (mIU/L) × mean fasting glucose (mmol/L)] ÷ 22.5. A HOMA-IR score of >2.0 has been demonstrated to accurately correlate with more invasive measures of insulin resistance, as determined through euglycemic clamps and intravenous glucose tolerance tests [27].

Controlled attenuation parameter (CAP) refers to an ultrasonographic coefficient that is affected by and directly proportional to the amount of liver fat. Consequently, CAP readings are commonly measured in conjunction with transient elastography, to quantitate hepatic steatosis. CAP values range from 100 to 400 dB/m, with higher values indicating a greater degree of liver fat. CAP measurement has now also been demonstrated to provide a further useful tool in the diagnosis and staging of NAFLD, with cut-off values > 268 dB/m accurately correlating with steatosis grades > 5–33% [25]. FibroScan-CAP measurements (performed at the discretion of the clinician) where available in the clinical notes were collated for review as part of outcomes before/after IGB insertion and part of the process for identifying obese patients with NAFLD who could be included into this analysis.

### 2.4. Statistical Analysis

Statistical analysis was performed using SPSS software (SPSS version 20.0, Chicago, IL, USA). Continuous variables were analysed using one-way ANOVA and nonparametric testing, while categorical variables were assessed via Chi-square and Fisher's exact analyses. Two-tailed *p* values of <0.05 were regarded as significant throughout.

## 3. Results

### 3.1. Baseline Characteristics

Follow-up data from a total number of 135 patients were available for analysis (Table 1). The majority were women (71%), of Caucasian background (66%), with a mean age of 47 ± 12 years. The mean baseline weight was 117.9 ± 22.0 kg (M: 122.7 kg; F: 115.7 kg), with baseline BMI of 41.7 ± 6.6 kg/m^2^ (M: 39.7 kg/m^2^; F: 42.8 kg/m^2^). Waist circumference measurements were also available in a subset of patients (*n* = 33), with an average recording of 124.2 ± 13.6 cm (accepted normal values being ≤94 cm for men and ≤80 cm for women). The majority of patients were insulin resistant, with a median HOMA-IR score of 3.6 and moderately abnormal liver function tests and adverse lipid profiles also apparent at baseline (Table 1). The majority of patients also had concurrent features of the metabolic syndrome (60/134, 45%), and 29% already required medical treatments for Type 2 diabetes (Table 1).

### 3.2. Clinical Outcomes at 6 Months (T1)

The mean time at IGB removal was 5.8 ± 1.7 months following insertion (Table 1). At the end of follow-up, significant reductions were seen in both weight and BMI for the majority of the cohort, with mean weight loss of 11.3 kg (117.9 kg to 106.6 kg, *p* < 0.01) and mean BMI reduction of 4.1 kg/m^2^ (41.7 to 37.6 kg/m^2^, *p* < 0.01), respectively (Table 2). In those with paired waist circumference measurements (*n* = 33), recordings also improved significantly following IGB therapy, 124.2 cm to 101.1 cm (mean reduction of 23.1 cm, *p* < 0.05).

Although there was a trend in the reduction of fasting serum lipids following IGB therapy, the results did not reach statistical significance (Table 2).

### 3.3. Clinical Outcomes following Serial IGB Therapy (T2 and T3)

**Figure 1:**
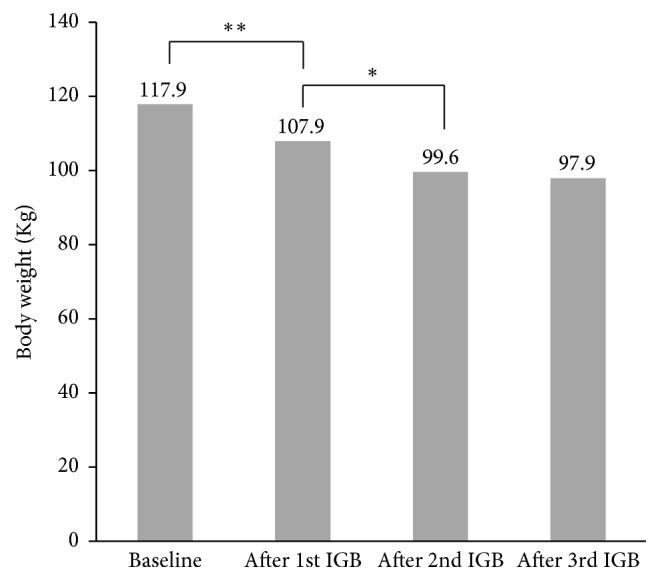
Incremental weight loss following serial IGB therapy (^*∗*^*p* < 0.05; ^*∗∗*^*p* < 0.01).

In this cohort of 135 patients, 67 had received only one IGB during the study period, while 48 patients received two serial IGBs, and 20 patients received three IGBs. The greatest amount of total weight loss was observed in the first 6 months after treatment with one IGB (T1), whereas the additional amount of weight loss achieved in those who received a 2nd (T2) and 3rd (T3) IGB were not as significant (mean weight loss of 7.4 kg versus 3.6 kg and 1.9 kg, resp.; *p* = 0.05) (Table 3). Furthermore, while the incremental weight decline following a second IGB was statistically significant, no further significant weight losses were observed in those who underwent treatment with a third IGB (Figure 1).

### 3.4. Changes in NAFLD Indices after IGB Treatment

After six months of IGB therapy, HOMA-IR scores were observed to significantly improve in a total of 78 patients with paired readings at baseline and final follow-up, 3.6 compared with 2.6 (*p* < 0.05). Significant improvements in serum ALT and GGT were also noted, but only when analysed in those with an elevated HOMA-IR score at baseline. The prevalence of elevated plasma ALT and GGT concentrations decreased from 42% to 22% and from 57% to 34%, respectively, after IGB therapy at six months (Table 2). Similar findings were noted with observed changes in BMI and ALT across the groups who received serial treatment with two or three IGBs, but no further significant reductions in HOMA-IR scores were observed (Table 3).

### 3.5. Long-Term Follow-Up after IGB Treatment

The average period of follow-up for the total cohort was approximately 20 months. During this time, 58%  (*n* = 78) of patients were lost to follow-up, and 20%  (*n* = 27) were eventually referred for bariatric surgery. Of those who continued in clinical follow-up for whom data were available (*n* = 37), the mean final weight and total % of baseline weight lost at final follow-up were 108.8 kg and 6.3% for T1, 107.5 kg and 9.0% for T2, and 115.3 kg and 7.5% for T3, respectively (*p* = 0.05).

### 3.6. Safety Outcomes

The majority of patients tolerated IGB therapy. The most common adverse symptoms reported were nausea and vomiting (20.7%) and abdominal cramps, experienced primarily in the first few weeks after IGB insertion. During this time period, only one patient required emergency balloon removal for an unexpected gastrointestinal obstruction [28], and 14 patients (10.4%) had their IGB removed prematurely for intolerance (Table 4). There were no predominant clinical factors that predicted IGB intolerance, including gender or age.

In the patient who experienced an unexpected obstruction, the IGB was found to be significantly distended with air and fluid while still within the stomach. Balloon puncture and retrieval were performed in the standard manner, with the patient making a swift recovery after balloon extraction. Prolonged cultures of the IGB fluid did not reveal any infective (gas-forming) organisms, which raised the possibility of a defective valve potentially allowing entry of air into the device, with consequent distension.

## 4. Discussion

Over the next decade, obesity and its related complications will continue to instigate overwhelming health-related population and cost burdens for most economies of the world [29]. Although there is increasing recognition of this burgeoning problem, more effective treatment modalities are still desperately needed. While lifestyle interventions, focusing on dietary changes and increased exercise, remain the primary treatment recommendation, endoscopic bariatric devices such as the IGB are fast becoming a viable option for improved management through assisted weight loss [30].

To date, several studies have reported on the efficacy of IGBs in inducing significant weight loss over the short to medium term [31, 32]. Similarly, we found that IGB therapy resulted in an average weight loss of 11.3 kg and BMI reduction of 4.1 kg/m^2^ over the initial 6 months, approximating to an average EWL (excess weight loss) of 22.5%. These findings are in keeping with figures reported by Imaz et al. [19] in their meta-analysis of several earlier studies examining the efficacy of IGB therapy, with pooled weight and BMI losses of 14.7 kg and 5.7 kg/m^2^, respectively.

In addition to the management of obesity and excess weight, IGB therapy appears to offer a promising adjunctive treatment modality for NAFLD/NASH, even independent of weight changes [21]. To date, only a few, relatively small studies have focused on the efficacy of IGB therapy in reducing hepatic steatosis. Forlano et al. [22] demonstrated a significant reduction in liver steatosis on serial abdominal ultrasonography in 91 “responder” patients who achieved significant weight loss after IGB treatment over 6 months. This correlated with a significant reduction in HOMA-IR scores (3.8 to 1.6, *p* < 0.001) and presumably improved insulin resistance. Similarly, Folini and colleagues [21] quantified improved hepatic steatosis on serial hepatic magnetic resonance imaging (MRI) in 18 patients who underwent IGB placement (or laparoscopic gastric banding) for 6 months, compared with no significant changes seen in 13 controls. Again, these changes correlated with improved BMI and significant reductions in weight and waist circumference.

Furthermore, in a prospective sham-controlled trial including 18 patients with obesity and NAFLD who underwent serial liver biopsies before and after 6 months of IGB placement, or sham therapy, liver histology demonstrated improved NAFLD Activity Scores (NAS) [33] in those who received IGB therapy versus no improvement in those who received sham treatment (intragastric normal saline solution infusion) [34]. However, no improvements in median lobular inflammation, ballooning, or fibrosis scores were seen in either group, although this may have been due to the short duration of treatment.

In our cohort, we also found improved insulin resistance through reduced HOMA-IR scores in those who achieved significant weight losses, which correlated with significant improvements in liver function tests (i.e., serum ALT and GGT) [4]. We also observed several cases of improved hepatic NASH fibrosis with IGB therapy, as measured though serial FibroScan recordings (results not shown) in a few patients and verified with serial liver biopsy results in one case. In this particular case, originally diagnosed with decompensated cirrhosis with portal hypertension from NASH, serial IGB treatment resulted in a total weight loss of 54 kg over 19 months, with improved portal inflammation and steatosis, and fibrosis remodelling thought to indicate early cirrhosis reversal on follow-up histology. To our knowledge, this case is the first to report on regression of cirrhosis following weight loss through nonsurgical means. This case is also more significant as it demonstrates how IGB therapy can provide an alternative weight loss tool for those with advanced liver disease, and often poor levels of medical and physical fitness, for whom treatment options are often severely limited.

This study is the first to document outcomes with serial (more than two) periods of IGB therapy. In their publication examining weight loss following two IGB treatments, Genco and colleagues [35] found improved and prolonged weight loss in 50 patients who underwent a second IGB following weight loss achieved with a primary balloon, as compared with control patients who were randomised to receive only dietary therapy following their initial IGB. In the group who received two serial IGBs, the mean BMI was 30.9 versus 35.9 kg/m^2^ (*p* < 0.05) in the control cohort at the end of approximately 13 months. In our analysis, we observed the greatest degree of weight loss following treatment with the 1st IGB (average weight loss of 7 kg posttherapy), while the amount of weight lost following the second and third IGBs was not as significant (mean weight loss of 3.6 kg and 1.9 kg, resp.), as compared with the start weights prior to each insertion period. Indeed, weight regain was actually observed in a few patients after their third IGB, which would suggest that optimal weight loss is likely achieved following two IGBs, over 12 months, while the third IGB may help to promote weight loss “maintenance” rather than further weight decline. Again, a greater magnitude of weight loss again correlated with the most significant changes in ALT, although we did not observe any concurrent improvements in HOMA-IR scores over the extended treatment period beyond the initial 6 months, likely due to the smaller degrees of weight change achieved during these later time points.

Although these results provide a promising outlook for short-medium term outcomes, the longer-term utility of IGB therapy still requires elucidation. Currently, there are few studies that report on the outcomes with IGBs beyond 12 months and none reporting on long-term outcomes with serial IGB treatments. In their report comparing weight loss outcomes on 130 patients who had retrospectively received IGB versus a matched cohort of controls who received only prospective, specialised dietary interventions, Genco et al. [36] found more significant maintenance of weight loss endpoints in the IGB group as compared with controls at both 6 months and 24 months. Similarly, a prospective analysis by Mitura and Garnysz [37] found that of 70 patients who received one IGB, at two-year follow-up, 45 patients still maintained their reduced weight, while 7 had returned to their baseline weight, and 18 patients had experienced a “yo-yo” effect with an average weight gain of 2.7 kg.

In our cohort, over an average longer-term follow-up period of 20 months, we found that 20% of patients were unable to maintain their initial weight loss, which necessitated a referral for bariatric surgery for most. The number of IGB treatments did not appear to significantly affect the long-term outcomes in this regard, although it should be noted that almost 50% of each group had chosen to discontinue their clinical follow-up during the study period. For those in follow-up who maintained their weight loss, this seemed to plateau at 12 months (with approx. 9% of baseline weight loss) and then decline following this period. While these results might suggest that the durability of weight loss outcomes with IGB treatment can be limited in the longer term, findings to date certainly demonstrate improved weight loss maintenance as compared with current standard of care (i.e., lifestyle interventions alone) and highlight the need for more prospective, controlled trials examining the efficacy of serial IGB therapy in the long-term treatment of obesity and NAFLD. Furthermore, it reinforces the important need for supplementary, holistic measures (psychological support) to optimise the management of obesity and other metabolic diseases.

The safety of IGB therapy has been verified in several earlier studies. Although our analysis revealed a 10% premature removal rate (due primarily to intolerance), which is higher than that reported in previous publications [19], the majority of patients underwent successful, uncomplicated insertions. The main side effects of nausea, vomiting, bloating, and abdominal cramping were reported to relent by 1–3 weeks following the point of insertion.

There are several limitations to our study. Firstly, this was an uncontrolled, retrospective review; therefore, we could not directly compare our outcomes with an untreated group, and some biochemical and anthropometric indices were not available in a substantive number of patients to enable a robust analysis on such changes beyond six months of follow-up. Furthermore, the number of patients who had received two and three serial IGBs was not comparable with those who had received only one IGB, which may have diminished the statistical significance of some outcome measures. Nonetheless, our results at six and twelve months are consistent with findings described in previous reports [19, 32]. Prospective studies examining longer-term outcomes, involving two or more serial IGBs, are indeed required to further validate these important endpoints and the potential utility of IGB therapy in the management of obesity and NAFLD.

In conclusion, obesity and its related complications, including NAFLD/NASH, will continue to present a major health burden if rates continue to rise over the next decade. Weight loss remains the primary treatment recommendation for most, but modalities to affect successful and durable weight loss are limited. While medical therapies are awaited, and bariatric surgery is not a viable option for all, endoscopic bariatric devices, such as the IGB, provide an alternative or adjunctive treatment with proven efficacy in the short-medium term, with an acceptable safety profile. More studies will be required to determine their potential role as a therapeutic weight loss option in the longer term and whether advanced metabolic liver diseases can definitely regress following effective treatment.

## Figures and Tables

**Table 1:** Baseline characteristics.

Clinical parameter	Patient numbers (*n* = 135)
Age (years), mean ± SD	47.1 (12.2)
Sex	M 39 (29%), F 96 (71%)
Ethnicity	
(i) Caucasian	89 (66%)
(ii) Black	27 (20%)
(iii) Asian (Indian)	2 (2%)
(iv) Asian (oriental)	2 (2%)
(v) Latino	6 (4%)
(vi) Mixed	3 (2%)
Diabetes	39/134 (29%)
Other MetSy features	60/134 (45%)
Taking ACE-inhibitor medication	94/133 (71%)
Fasting BSL [*n* = 96] (mmol/L), mean ± SD	6.0 ± 2.7
Weight [*n* = 134] (kg), mean ± SD	117.9 ± 22.0
BMI [*n* = 96] (kg/m^2^), mean ± SD	41.7 ± 6.6
Waist circumference [*n* = 33] (cm), mean ± SD	124.2 ± 13.6
Fasting cholesterol [*n* = 50] (mmol/L), mean ± SD	4.8 ± 1.4
Fasting LDL [*n* = 41] (mmol/L), mean ± SD	2.7 ± 1.1
Fasting HDL [*n* = 41] (mmol/L), mean ± SD	1.2 ± 0.3
Fasting triglycerides [*n* = 41] (mmol/L), mean ± SD	1.8 ± 1.0
Fasting insulin [*n* = 83] (mIU/L), mean ± SD	136.0 ± 169.0
HOMA-IR [*n* = 78], median (range)^*∗*^	3.6 (2.1–5.9)
ALT [*n* = 108] (IU/L), mean ± SD	38.9 ± 30.6
AST [*n* = 16] (IU/L), mean ± SD	35.1 ± 25.2
GGT [*n* = 82] (IU/L), mean ± SD	62.6 ± 74.9
Fib-4 score [*n* = 12], mean ± SD	1.5 ± 0.7
Period of 1st IGB placement (months), mean ± SD	5.8 ± 1.7
Total number of balloons	One = 135, two = 48, three =20

^*∗*^25–75% interquartile ranges expressed.

**Table 2:** Clinical outcomes at 6 months.

Clinical parameter	Baseline	6 months (after IGB removal)	Mean difference	*p* value
*Weight (kg)*	*117.9*	*106.6*	*11.3*	*<0.01*
*BMI (kg/m* ^*2*^)	*41.7*	*37.6*	*4.1*	*<0.01*
*Waist circumference (cm)*	*124.2*	*101.1*	*23.1*	*0.04*
Fasting BSL (mmol/L)	6.0	5.4	0.6	0.12
Fasting insulin (mIU/L)	136.0	96.5	39.5	0.12
*HOMA-IR* ^*∗*^	*3.6*	*2.6*	*1.0*	*0.03*
*ALT (IU/L)* ^*∗*^	*38.9*	*31.0*	*7.9*	*<0.01*
AST (IU/L)	35.1	32.8	2.3	0.11
*GGT (IU/L)* ^*∗*^	*62.6*	*39.1*	*23.5*	*<0.01*
Fasting cholesterol (mmol/L)	4.8	5.1	−0.3	0.08
Fasting LDL (mmol/L)	2.7	2.8	−0.1	0.09
Fasting HDL (mmol/L)	1.2	1.5	−0.3	0.39
Fasting triglycerides (mmol/L)	1.8	1.4	0.4	0.22

^*∗*^A significant difference in ALT and GGT was noted at follow-up in those with an elevated HOMA-IR score (i.e., insulin resistance) at baseline.

**Table 3:** Anthropometric and ALT changes following serial IGB therapy.

Clinical parameter	After 1st IGB (T1) [*n* = 67]	After 2nd IGB (T2) [*n* = 48]	After 3rd IGB (T3) [*n* = 20]	*p* value
Final weight (kg)	107.9 ± 23.5	99.6 ± 17.1	97.9 ± 43.4	0.18
Incremental weight change^*∗*^	−7.4	−3.6	−1.9	0.05^§^
Total weight change^*∗∗*^	−6.9	−10.6	−9.4	<0.01^§^
Final BMI (kg/m^2^)	39.0	35.5	38.2	0.15
Total change in BMI^*∗∗*^	−2.0	−5.7	−5.5	<0.01^§^
Final HOMA-IR	4.27	1.89	2.71	0.21
Total change in HOMA-IR^*∗∗*^	−1.17	−1.37	−3.00	0.71
Final ALT (IU/L)	31.7	27.9	26.3	0.57
Total change in ALT^*∗∗*^	−5.8	−4.2	21.8^¶^	<0.01^§^
Total follow-up (months)	17.1	18.8	26.6	
Final outcomes:				
(i) Loss to follow-up	44	25	9	0.65
(ii) Referred for surgery	12	10	5	
(iii) Still in active follow-up	11	13	6	

^*∗*^Changes compared with the start weight prior to IGB insertion, and the end of the treatment period (T1/T2/T3).  ^*∗∗*^Changes compared with baseline (prior to placement of the 1st IGB), and final follow-up.  ^¶^Data only available in 5 cases.  ^§^Significant changes were observed in outcomes between T1 and T2, but not between T2 and T3.

**Table 4:** Adverse outcomes.

Side effect	Number of patients
Nausea and vomiting	28 (20.7%)
Abdominal pain	8 (5.9%)
Abdominal bloating/flatulence	16 (11.9%)
Constipation or diarrhoea	6 (4.4%)
Gastroesophageal reflux	9 (6.7%)
Erosive gastritis or oesophagitis	2 (1.5%)
Deflation or displacement of IGB	1 (0.7%)
Gastrointestinal obstruction	1 (0.7%)
Premature removal	15 (10.4%)
